# Do psychological capital and transformational leadership make differences in organizational citizenship behavior?

**DOI:** 10.1371/journal.pone.0294559

**Published:** 2023-12-15

**Authors:** Heni Yuwono, Muhammad Danang Kurniawan, Nanank Syamsudin, Anis Eliyana, Deddy Eduar Eka Saputra, Alvin Permana Emur, Nurul Iman Abdul Jalil

**Affiliations:** 1 Directorate General of Corrections, Ministry of Law and Human Rights of the Republic of Indonesia, Central Jakarta, DKI Jakarta, Indonesia; 2 Department of Management, Universitas Airlangga, Surabaya, East Java, Indonesia; 3 Research and Publication, PT Usaha Mulia Digital Indonesia (PT UMDI), South Jakarta, DKI Jakarta, Indonesia; 4 State Development Administration, Politeknik STIA LAN Jakarta, Central Jakarta, DKI Jakarta, Indonesia; 5 Postgraduate School, Universitas Negeri Jakarta, East Jakarta, DKI Jakarta, Indonesia; 6 Department of Management, Universitas Indonesia, Depok, West Java, Indonesia; 7 Department of Psychology and Counseling, Universiti Tunku Abdul Rahman, Kampar, Perak Darul Ridzuan, Malaysia; National University of Sciences and Technology, PAKISTAN

## Abstract

This research is proposed to determine factors affecting organizational citizenship behavior (OCB), tested on counselors, totaling 156 respondents. This study applied three waves in data collection with an interval of 30 days and a multigroup analysis to validate OCB. The analysis technique used is the Structural Equation Modeling (SEM) method using the IBM SPSS AMOS v26. The results showed that transformational leadership and psychological capital could, directly and indirectly, influence OCB and substantially affect work engagement as the mediator. Furthermore, male counselors with OCB were more dominantly influenced by work engagement, whereas female counselors were by transformational leadership. The results of this study can be used as a basis for policy recommendations by organizational management, especially organizations in the public service. This research has strengthened the empirical foundation on voluntary extra-role behavior and initiatives that can improve organizational effectiveness. This behavior can be manifested by strengthening psychological capital, transformational leadership, and work engagement.

## 1. Introduction

Organizations can survive and grow in today’s world if they use their human resources best [1]. As a result, organizations must identify variables that inspire employees to volunteer for activities that are not directly related to their tasks. If workers successfully carry out their responsibilities, it will ultimately improve the overall quality of the organization [2]. In general, individual behavior in an organization is directed to support the effectiveness and efficiency of the organization [3]. One individual behavior that is considered capable of supporting this is Organizational Citizenship Behavior (OCB). OCB is the voluntary behavior of employees in addition to the tasks assigned to them that contribute to the overall effectiveness of an organization [2]. Hence, OCB can become a behavior that is beneficial for organizational productivity.

The success of an organization must be connected to the critical role of human resources, which also applies to correctional institutions in Indonesia. The biggest challenge currently faced by correctional institutions is overcrowding. According to data from the Correctional Database System of the Directorate General of Corrections in 2022, there were 270,780 prisoners occupying correctional units with a capacity of 135,568 throughout Indonesia [4]. In addition, correctional institutions in Indonesia generally have fewer counselors than convicts, so they face big challenges in providing the best service for convicts [5]. It shows how counselors must go beyond their proper job description and how voluntary behavior from counselors is needed to reduce the burden that correctional institutions must bear. This can take the form of assisting coworkers or superiors outside the scope of one’s job description. As part of individual performance, it is anticipated that the existence of OCB will be able to contribute to the operation of correctional institutions, which are associated with organizational performance [6].

Individuals need to be satisfied with their employment and earning money [7]. As a result, it is critical to figure out how people might be happier, more successful, and more content in their careers. The state of positive psychological development of an individual is referred to as psychological capital [8]. Positive psychology is a notion that heals the bad aspects of people’s lives while focusing on their happiness and helping them grow [7]. Psychological capital is an individual’s good psychological state linked to organizational variables like OCB [9]. Psychological capital is considered an important personal resource that can facilitate counselors in completing and achieving targets well [1]. The important resource is realized through optimism for a better situation despite the organization’s uncertainty. The complex phenomena in organizations need more attention in order to take the appropriate approach to improve individual performance by considering individual differences [10], such as psychological capital as one of the main determinants of OCB for organizational performance. Counselors with strong psychological capital have the desire and motivation within themselves to increase productivity at work and complete tasks as well as possible. As a result, the counselors’ psychological capital is critical in ensuring they are satisfied and productive employees in their professional lives.

As human resources, employees are the most important asset owned by an organization because its success can be seen from the aspect of its employees and leadership [11]. Leadership can impact OCB behavior since an organization will collapse without good leadership. According to a previous study, transformational leadership is one of the leadership styles that might directly lead to OCB behavior [12, 13]. There is widespread agreement that transformational leadership can motivate workers to pursue a common cause despite their bigger self-interest [14]. Nonetheless, the impact of transformational leadership on OCB and its mechanisms in public institutions has not been revealed by previous studies [15]. Therefore, this study focuses on the link between transformational leadership and OCB in public organizations (correctional institutions). The contribution of transformational leadership to OCB can be explained theoretically with Social Exchange Theory (SET) which emphasizes reciprocity due to interactions between two parties [16]. This interaction is initiated by transformational leaders who provide support to followers so that followers respond with OCB as a form of responsibility. Furthermore, transformational leadership is important for correctional institution because good transformational leaders can reduce tension and encourage staff motivation to complete work well, even outside their duties and functions.

Occupational psychologists believe OCB is increasingly prevalent because engaged individuals simultaneously invest their cognitive, physical, and emotional resources in their job [1]. In the last two decades, work engagement has become a prominent and fascinating topic in management and positive psychology [17]. Participation of correctional counselors is advantageous for the organization since it is related to positive organizational outcomes that promote its effectiveness.

A number of prior studies have demonstrated solid empirical support for the contribution of organizational, individual, and labor factors to OCB. [15]. Those factors include psychological capital [18, 19], transformational leadership [20, 21], and work engagement [22, 23]. Other researchers have revealed that work engagement mediates the influence of psychological capital on OCB [1]. However, these three antecedents have not been fully studied in the context of public institutions [15]. Thus, it aims to examine the mediating role of work engagement in supporting transformational leadership and psychological capital as antecedents of OCB correctional institutions. It is important to reveal the individual and organizational mechanisms that support OCB in the context of public institutions which have not received much attention from previous research.

## 2. Literature review

### 2.1 Conceptual basis

#### 2.1.1 Psychological capital

Psychological capital is defined as a positive development of an individual’s psychology manifested through positive behavior, such as: being optimistic that problems will be solved, believing that everyone can be successful, leading new paths when necessary to achieve goals and targets, and ensuring the organization’s continued success [6]. Furthermore, psychological capital can generate positive emotions, which employees can use to share creative ideas or make enhancement suggestions [2]. Psychological capital is a valuable resource that influences many facets of an individual’s existence, including motivation, emotion, cognition, and behavior. It assists individuals and organizations in developing competitive advantages beyond human and social capital [10]. In this sense, psychological capital is a form of self-efficacy, resiliency, optimism, and hope that is extraordinarily useful for generating other resources associated with desired employee behavior.

#### 2.1.2 Transformational leadership

Transformational leadership may motivate employees or followers to transform their values, beliefs, abilities, and motives to reach better organizational performance by instilling a feeling of self-motivation in them [24]. Transformational leadership is also defined as a process in which a leader serves as an ideal role model, encourages creative behavior, supports and directs people, and motivates them to reach a shared vision and set of goals [20]. Transformational leadership focuses on employee development by evaluating employees’ potential in performing their work and observing the possibility of extending their future responsibilities [25]. Thus, transformational leadership is necessary for explaining the efforts made by employees motivated by leaders with transformational to perform beyond the expected level of performance.

#### 2.1.3 Work engagement

It should be noted that in research, the phrases "employee engagement" and "work engagement" have been used interchangeably [26]. This study uses "work engagement" to describe employees’ relationship with their job. It is defined as a positive, high-energy condition marked by high devotion and a strong focus on absorption in the workplace [26]. Work engagement demonstrates passion in maintaining high energy and mental resilience in the face of adversity. At the same time, dedicated individuals have a sense of enthusiasm and confidence in challenging things and have a strong personal identification with work [24]. At work, vigor is defined as a person’s more significant energy and psychological resilience; commitment is pride, enthusiasm, motivation, and challenge; and absorption is defined as someone who can concentrate thoroughly enough to refuse to quit working [27]. Thus, work engagement shows employees who have invested physical and psychological energy to pursue individual and team goals by being willing to invest effort and stay on the job.

#### 2.1.4 Organizational Citizenship Behavior (OCB)

OCB is voluntary employee behavior that helps the organization function without difficulty [21]. OCB is independent individual behavior since the formal reward system does not directly or openly acknowledge it and supports organization effectiveness [23]. OCB, also known as extra-role behavior, occurs when employees go beyond their job descriptions for intrinsic reasons [11]. OCB might require working extra hours, promoting goodwill and institutional image, reducing organization’s excessive expenses, adhering to organizational standards, and preserving a strong work ethic [14]. Another study found multiple OCB-promoting variables which comprise of job, employee, and organizational characteristics [13]. Thus, OCB is a sort of voluntary behavior that helps organizations by showing generosity and concern for others by performing extra tasks.

### 2.2 Hypothesis development

#### 2.2.1 Psychological capital and Organizational Citizenship Behavior (OCB)

Psychological capital can assist individuals in achieving work-related goals and inspire extra-role behavior [1]. Psychological capital is a crucial antecedent of prosocial behavior, demonstrating that employees with high well-being are likelier to display OCB than those with poor well-being [2]. According to previous research, psychological capital affects and drives OCB [9]. Employees with solid psychological capital feel competent in obtaining a job by realizing their talents, which makes them more hopeful about the future without fear since they are more optimistic about unpleasant things [7]. An employee with psychological capital will have suitable activities that indicate OCB towards the company and the person. Psychological capital can show OCB to coworkers and pro-active role behavior to their organization [1]. Psychologically healthy counselors will want to support their coworkers freely and manage conflicts properly to implement OCB. This study thus suggests a hypothesis.

H1: Psychological Capital has a significant effect on Organizational Citizenship Behavior (OCB)

#### 2.2.2 Psychological capital and work engagement

Psychological capital will benefit work engagement because individuals with psychological capital can have energy at work, encouraging them to exchange knowledge with coworkers more freely and readily [8, 28]. Additionally, they can demonstrate passion at work, which leads to favorable results such as work engagement. This remark is supported by previous research, which indicates that persons with psychological capital tend to function as contextual resources to invest in their job [29]. By responding positively, individuals with stable self-efficacy, optimism, hope, and resiliency can maintain their attention on the task at hand and attribute errors to external environmental situations instead of their characteristics [29]. Individuals with psychological capital are believed to have abundant psychological resources and good emotional states, especially when presented with work-related obstacles, and are constantly optimistic. Psychological capital enhances work engagement by combining resilience, determination, and concentration [11]. Therefore, counselors with psychological capital can enhance their team’s morale and transmit pleasant feelings, motivating them to exert more significant effort in their tasks. In addition to the significance of psychological capital for employee well-being and organizational success, the previous study found that its association with work engagement is still restricted [30].

H2: Psychological Capital has a significant effect on Work Engagement.

#### 2.2.3 Transformational leadership and Organizational Citizenship Behavior (OCB)

A transformational leader can improve employee awareness and knowledge of moral principles and create visions that can drive employees to go beyond their aims and interests for the common good, such as the additional function of OCB [31]. Furthermore, transformational leadership is crucial for achieving exceptional role performance, such as OCB. This idea is supported by previous study assertions that transformational leadership can encourage employees to go beyond personal interests to pursue a joint mission, namely the superlative performance of the organization [20]. In return, an employee will demonstrate OCB. Additionally, prior empirical study has demonstrated a strong correlation between transformative leadership and OCB [20]. Transformational leadership will affect the expansion of OCB since it might inspire people to better their performance and hence get more interested in OCB [12, 13]. Additionally, transformational leadership involves fundamental adjustments to employees’ objectives, beliefs, and ambitions so that individuals are naturally driven to enhance performance beyond their job descriptions due to conformance with their values. In other words, transformational leaders have a tendency to support followers in carrying out their work as a form of positive treatment from leaders to their followers. In this case, transformational leadership can be seen as a form of organizational support for individuals. In line with SET, this good treatment will also be well-reciprocated by followers [32] in the form of OCB [33]. Previous research indicates that organizational support will generate a favorable likelihood among individuals to assist the organization [34]. In this case, OCB is a form of followers’ responsibility to their transformational leader. Therefore, transformational leadership is a crucial factor that promotes good behavior and learning among counselors undertaking work outside their responsibilities, leading to OCB.

H3: Transformational Leadership has a significant effect on Organizational Citizenship Behavior (OCB)

#### 2.2.4 Transformational leadership and work engagement

By promoting intrinsic motivation to aid in high-level leader-member interchange, transformational leadership is recognized to manage to meet and develop the high-level requirements of its employees. It has a more considerable effect on work engagement [11]. Transformational leadership, in particular, can foster a supportive organizational atmosphere that encourages high levels of work engagement by providing intellectual stimulation and personalized consideration [17]. Employees who believe their leader meets their need for advancement should find purpose in their work, and as a result, they will feel compelled to raise their work engagement [24]. Employees who perceive individualized transformational leadership consideration must also deal with the psychological demands of the workplace, which must allow them to be psychologically available to engage in their work thoroughly. Employees will be more inclined to work harder to accomplish the goal established by these leaders through realizing work engagement. As we know, transformational leadership is motivating and visionary. Previous studies have suggested that transformational leadership significantly affects work engagement [17, 24]. Thus, transformational leadership can set clear expectations and appreciate counselors for the good, fair, and considerate performance of those who bring feelings of engagement to work and psychological security.

H4: Transformational Leadership has a significant effect on Work Engagement

#### 2.2.5 Work Engagement and Organizational Citizenship Behavior (OCB)

Employees who demonstrate work engagement will tend to be focused, attentive, and emotionally involved with their work, making them more likely to leave their formal role in the organization and demonstrate OCB [35]. Work engagement is a positive resource that leads to high levels of motivation among employees, regardless of their responsibilities [35]. Highly motivated employees can perform their tasks effectively and efficiently, expanding their resource capabilities and encouraging OCB [36]. Work engagement is a concept that shows the commitment of cognitive, mental, and physical energy to work with someone who tends to be very energetic and motivated to work extra and is more likely to increase their contribution by doing more OCB [26]. Work engagement has been demonstrated to be a constant and robust predictor of OCB in a recent study [23]. This investigation has advised that studies be conducted to conclude the causal association between work engagement and OCB. Counselors engaged at work can demonstrate OCB because they perform their assignments quickly and feel capable of taking on additional responsibility.

H5: Work Engagement has a significant effect on Organizational Citizenship Behavior (OCB)

#### 2.2.6 Mediating role of work engagement

Previous research has proposed that psychological capital predicts work engagement and that, when presented with a challenge, persons with psychological capital are more likely to feel in control of the issue, making them more eager to begin working immediately [11]. These engaged employees will also be loyal to organizations that make them happy. In addition, workers will feel compelled to reciprocate the advantages they get from their employer to foster similar ideals and a supportive atmosphere, which will lead to the additional function of OCB [1]. Feelings of psychological capital can motivate engaged people to work harder and contribute to the growth of their enterprises. By doing so, it is anticipated that they will exhibit more OCB than their coworkers. Prior research demonstrates that employees can only exhibit voluntary work behavior toward the organization if they are committed to their work. Employees must find the job exciting and assume responsibility [1]. Therefore, recent research has constructed a theoretical framework for work engagement that mediates the relationship between psychological capital and OCB among working counselors.

H6: Work Engagement significantly mediates the effect of Psychological Capital on Organizational Citizenship Behavior (OCB)

Research on the role of work engagement as mediator of transformational leadership on OCB shows that leaders can involve creative, innovative subordinates and are proactive in behaving OCB [35]. The work engagement that counselors have for the task given will be more pronounced when they get positive support from transformational leaders. High work engagement will also have an impact on the counselors’ behavior who take the initiative and voluntarily to increase organizational effectiveness even though it will be indirectly related to the organization’s formal reward system, such as OCB. However, it will indirectly be related to the organization’s formal reward system, such as OCB. The role of individuals in being loyal to their work can mediate the effect of transformational leadership on OCB [12, 13]. It means that if transformational leadership is applied consistently by the expectations of counselors to the leader, then employees will produce work engagement and will do something from their work but are also willing to work outside of their duties, such as OCB.

H7: Work Engagement significantly mediates the effect of Transformational Leadership on Organizational Citizenship Behavior (OCB)

**Fig 1:**
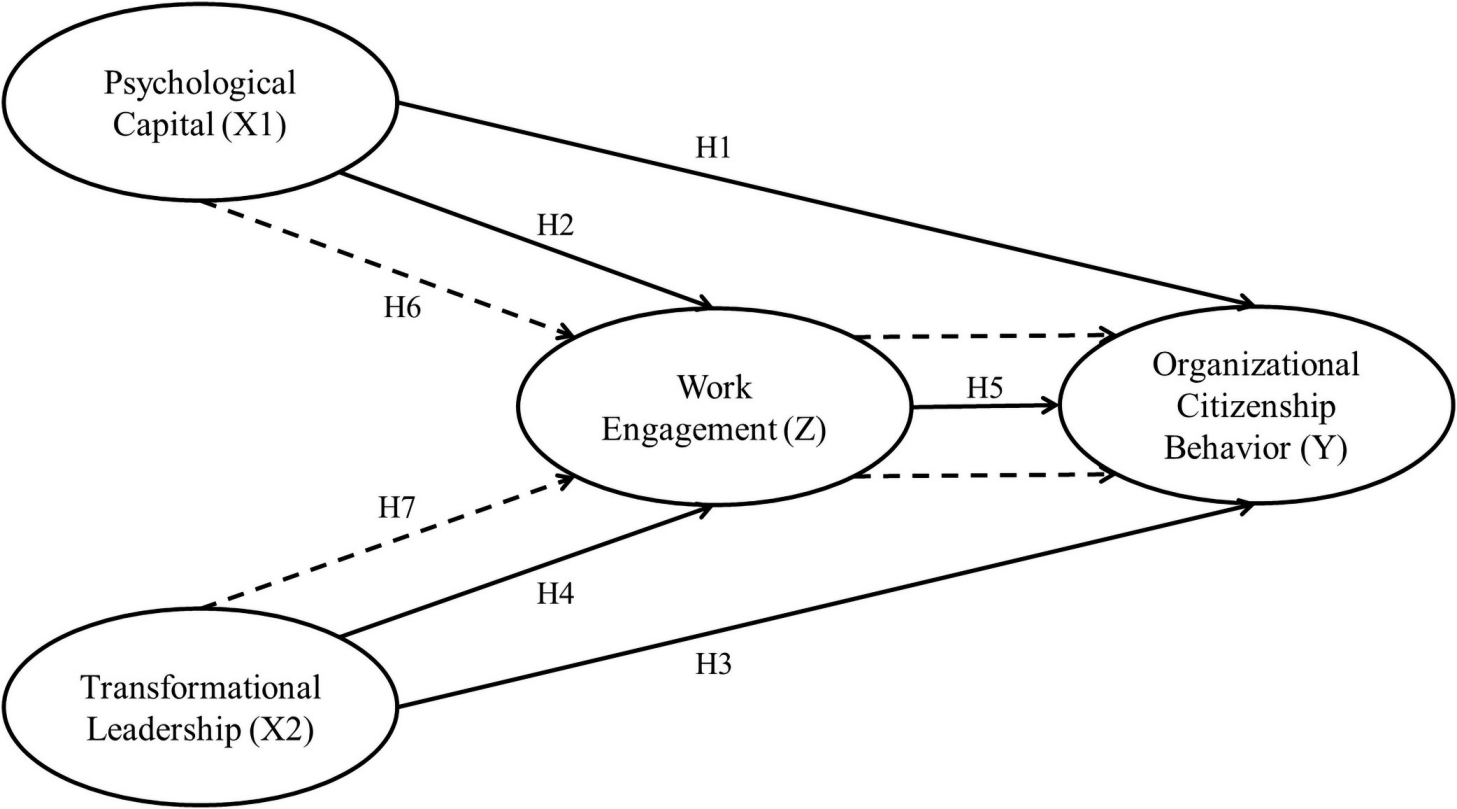
Conceptual framework.

The hypotheses are conceptualized in Fig 1 below.

## 3. Research methods

### 3.1 Data collection

The population in this study were all client counselors in Indonesia, as many as 1565 counselors. Meanwhile, the sampling was selected using a purposive sampling technique. Purposive sampling is a technique in which the researcher determines the sample based on certain criteria [37]. The selection criteria were: (1) client counselors who have direct interaction with the terrorism convicts and (2) provide guidance and counseling for terrorism convicts. After filtered with the purposive sampling technique, the remaining numbers of counselors were 156. Counselors who are responsible for terrorism convicts are chosen because of their vital role in suppressing serious and high-risk crimes. Furthermore, data collection was carried out by distributing online questionnaires for three distributions with a span of 30 days each. The first distribution was carried out to measure psychological capital and transformational leadership. The second one was carried out to measure work engagement. Lastly, OCB is measured on the third distribution. The results of distributing the questionnaires are in previous publication which is an integral part of this study [38].

Since this is a non-interventional study, the Development and Innovation Institute of Publishing Journal and Intellectual Property Rights at Universitas Airlangga (LIPJPHKI) ruled that no ethical approval was necessary. Following the organization’s policy, the chief executive, who represented the organization, also granted written informed consent. LIPJPHKI also validated the consent. In addition, the participants agreed prior to questionnaire completion that the information would be kept confidential and utilized just for research purposes.

### 3.2 Measurement

The independent variables used in this research are psychological capital (X1) and transformational leadership (X2), with the dependent variable being OCB (Y). In addition, this study also used a mediating variable, namely work engagement (Z). The data was collected by distributing online questionnaires via Google Forms. The measuring scale utilized a Likert scale ranging from 1 (strongly disagree) to 5 (strongly agree). In measuring variables, this research adopted instruments developed by previous studies. A total of 20 items were used to measure transformational leadership [39]. Furthermore, 12 items measured psychological capital [40]. Work engagement was measured by 16 items [41]. Meanwhile, OCB was measured by 15 items [42]. All variable measurements in this study are listed in the Appendix.

### 3.3. Data analysis techniques

This research used the multivariate analysis method to test several variable relationships. The analytical technique was the structural equation modeling (SEM) method using the IBM SPSS AMOS application. SEM was intended to determine the influence magnitude of independent variables on dependent variables.

### 3.4 Robustness

The data for this study were collected in three waves. Three data waves were obtained with a 30-day interval between them. Although all variables were assessed on different time waves, this study aimed to reach findings that eliminate the risk of erroneous conclusions. In addition, this research simultaneously analyzed the constructs’ validity and reliability and compared two identical male and female models using multigroup analysis.

## 4. Results and discussion

### 4.1 Result

The respondents in this study were primarily men (60.3 percent), married (81.4 percent), aged 31–40 years (42.9 percent), possessed a bachelor’s degree (70.5 percent), and had worked more than eight years (71.8 percent). They became civil servants through the general test (96.2 percent) and were in functional positions (100 percent).

According to the description results of psychological capital, transformational leadership, work engagement, and OCB (agree), the average score for this study was between 3.44 and 4.22, indicating that client counselors in Indonesia have these qualities.

**Fig 2:**
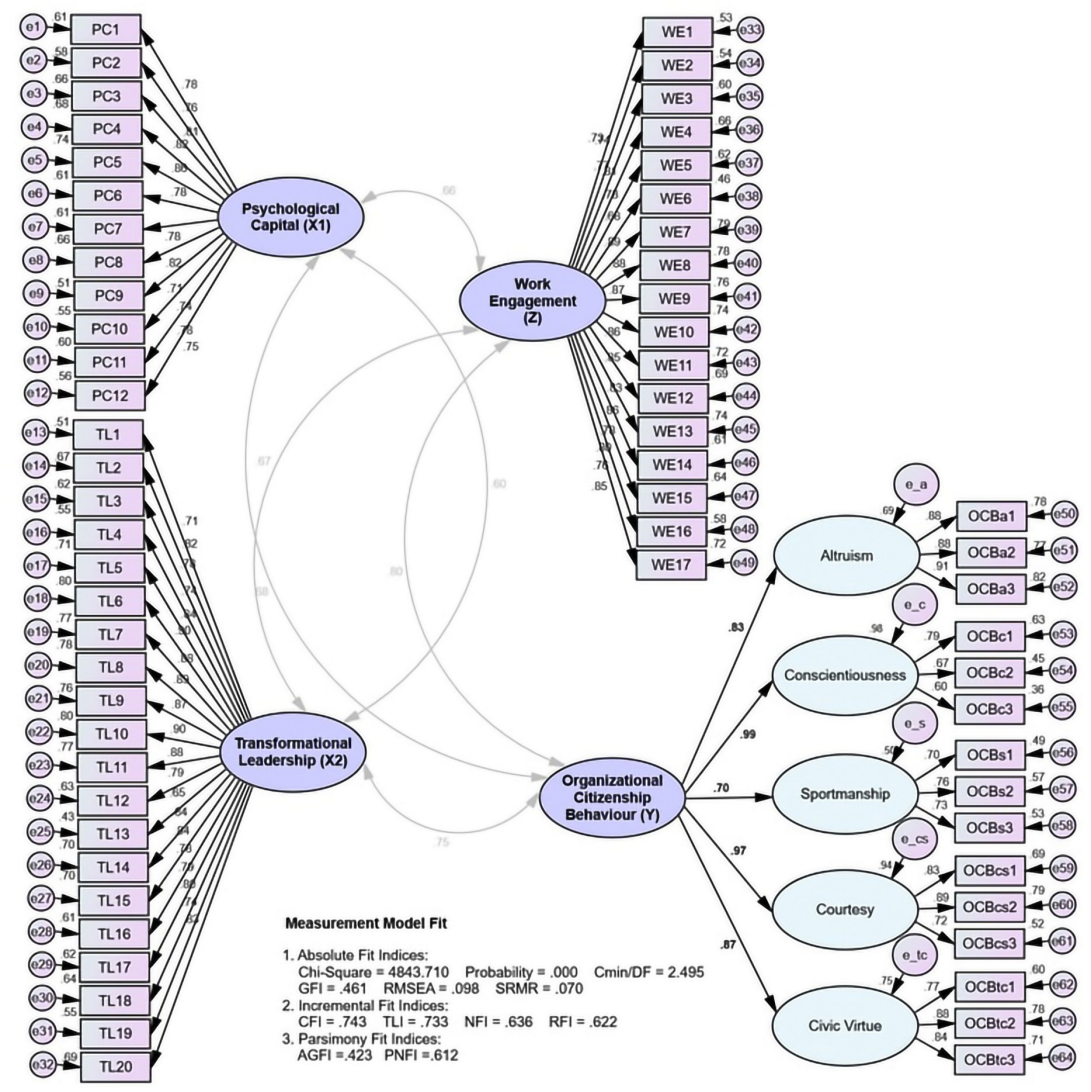
Assessing the measurement model.

**Fig 3:**
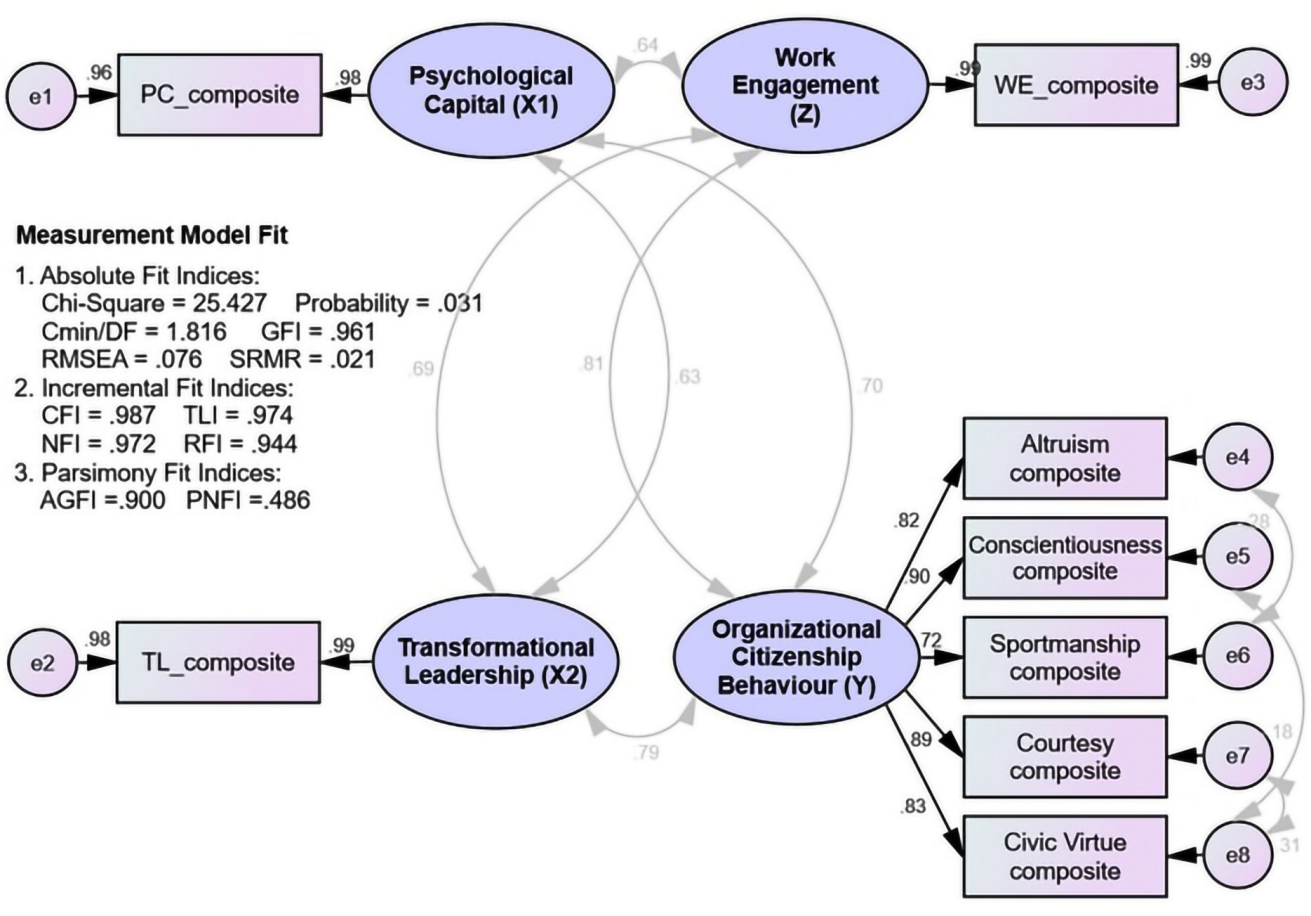
Assessing the measurement model (composite indicators).

The results of the estimation of the suitability of the measurement model are presented below:

The summary of the measurement model’s suitability test (Fig 2) revealed that the results of the original model’s suitability evaluation still produced requirements for absolute and incremental fit indices that had not been accepted (poor fit). Thus, the measurement model could not be accepted and needed revision. Furthermore, the model was improved using the composite indicators approach (Fig 3) and modifying the model guided by the modification indices. The suitability evaluation of the amended model revealed criteria for absolute and incremental fit indices that were all acceptable (good fit), indicating that the measurement model was suitable and could be used for further analysis.

Then in the evaluation results of construct validity, it was shown that the measurement model of all indicators produced a factor loading value of more than 0.50. Thus, all of them were valid for measuring psychological capital constructs, transformational leadership, work engagement, and OCB and could be used to build the structural model.

Table 1 demonstrates that each construct has a construct dependability value greater than 0.70 and an AVE value greater than 0.50, implying that the indicators are reliable in representing psychological capital, transformational leadership, work engagement, and OCB.

**Table 1:** Construct reliability.

Construct	Construct Reliability	Average Variance Extracted	Decision
Psychological Capital (X1)	0.950	0.615	Reliable
Transformational Leadership (X2)	0.975	0.665	Reliable
Work Engagement (Z)	0.970	0.657	Reliable
Organizational Citizenship Behavior (Y)			
Altruism	0.918	0.789	Reliable
Conscientiousness	0.755	0.509	Reliable
Sportsmanship	0.772	0.531	Reliable
Courtesy	0.856	0.667	Reliable
Civic Virtue	0.873	0.698	Reliable

**Fig 4:**
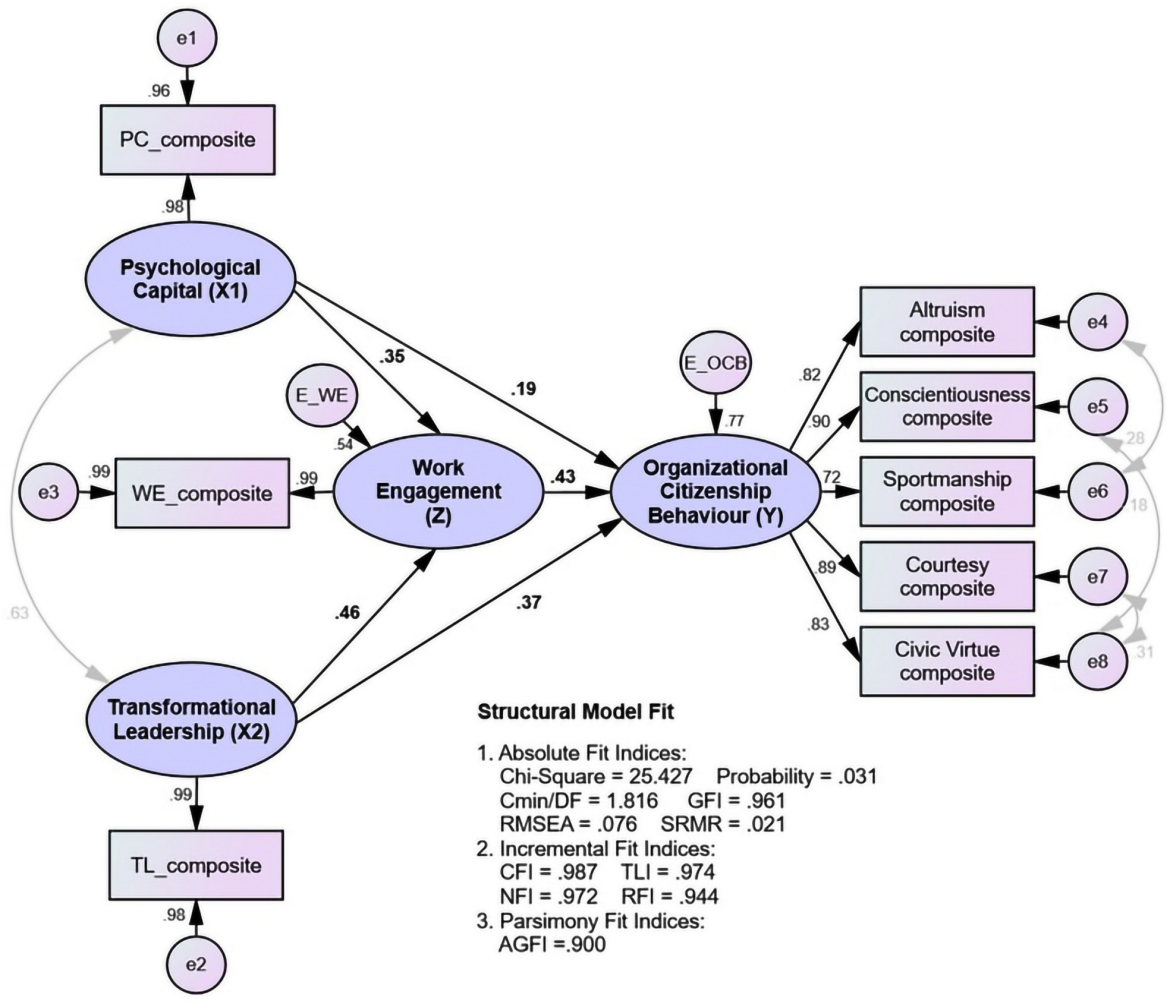
Assessing the structural model.

The calculation results of the goodness of fit indices generated by the structural model are presented in Fig 4:

Table 2 shows that all the absolute and incremental fit indices criteria have been met (good fit), indicating that the structural model has been accepted. The significance of the influence between variables, both direct and indirect effects, was then tested.

**Table 2:** Fit measure for the structural model.

Fit Measure		Critical Value	Structural Model	Decision
Absolute Fit Indices	Probability	> 0.05	0.031	Even good fit
	Cmin/DF	2.00	1.816	Good fit
	GFI	0.90	0.961	Good fit
	RMSEA	0.08	0.076	Good fit
	SRMR	0.08	0.021	Good fit
Incremental Fit Indices	CFI	0.95	0.987	Good fit
	TLI	0.95	0.974	fit
	NFI	0.90	0.972	Good
	RFI	0.90	0.944	Good fit
Parsimony Fit Indices*	AGFI	0.90	0.900	Good fit

* is not used for testing the suitability of a single model

The outcomes of structural testing relationships used to assess each research hypothesis based on SEM output are as follows:

Table 3 presents structural relationships testing, which uses the critical ratio (CR) and the probability value to test the hypothesis of the importance of the influence between variables (p-value). It is decided that there is a significant effect between these variables utilizing the provisions of the CR 1.96 or the p-value 5 percent significance level. So that all research results show that all hypotheses formulated can be accepted.

**Table 3:** Summary of the direct effect hypotheses.

Structural relationship	Std. Estimate	P-value^(^^a^^)^	Hypothesis
Psychological Capital → OCB	0.191	0.007**	H_1_ accepted
Psychological Capital → Work Engagement	0.351	0.006**	H_2_ accepted
Transformational Leadership → OCB	0.369	0.025*	H_3_ accepted
Transformational Leadership → Work Engagement	0.463	0.019*	H_4_ accepted
Work Engagement → OCB	0.432	0.002**	H_5_ accepted

*. Significant at the 0.05 level

**. Significant at the 0.01 level

n.s. Not significant

^(a)^ p-value has been calculated based on MLE bootstrapping.

The nature of mediation is determined by its mediating effect. If the direct effect of the exogenous variable on the endogenous variable is significant, and the effect through the mediating variable is also significant, then it is partially mediated. Conversely, if the direct effect is insignificant, while the indirect effect is significant, it is said to be fully mediated or perfect mediation. Table 4 shows that all indirect relationships are partial mediation.

**Table 4:** Summary of the indirect effect hypotheses.

Structural relationship	Std. Estimate	P-value^(a)^	Hypothesis	Type of mediator
Psychological capital → Work Engagement → Organizational Citizenship Behaviour	0.152	0.001**	H_6_ accepted	Partially mediation
Transformational leadership → Work Engagement → Organizational Citizenship Behaviour	0.200	0.003**	H_7_ accepted	Partially mediation

*. Significant at the 0.05 level

**. Significant at the 0.01 level

n.s. Not significant

^(a)^ calculated based on MLE bootstrapping

Analyses of coefficient of determination (R2) and effect size (f2) were performed to ensure that the model could adequately explain the empirical phenomena observed in the data [43]. R2 indicates the exogenous variables’ ability to explain endogenous variables. According to Table 5, the adjusted R2 value of work engagement is 0.532. This value indicates that psychological capital and transformational leadership can be explained by the 52.3% work engagement of the counselor. Using the same logic, counselor OCB can be explained by psychological capital, transformational leadership, and work engagement at 67.4%. Accordingly, this research model can explain OCB among correctional counselors based on empirical phenomena.

**Table 5:** Coefficient of determination.

Endogenous Variables	R^2^ Adjusted
Work Engagement	0.532
Organizational Citizenship Behavior	0.674

Next, effect size analysis was carried out by referring to the f2 value. The f2 value indicates changes in the R2 value if certain exogenous variables are removed from the model. Based on Table 6, the f2 value of psychological capital and transformational leadership on work engagement is in the middle category (0.15 > f2 > 0.35). The effect size of transformational leadership and work engagement on OCB is also in the medium category. On the other hand, the effect size of psychological capital on OCB is in the small category (0.02 > f2 > 0.15) [44].

**Table 6:** Effect size.

Variables	Work Engagement	Organizational Citizenship Behavior
Psychological Capital	0.241	0.062
Transformational Leadership	0.254	0.182
Work Engagement	-	0.253

**Fig 5:**
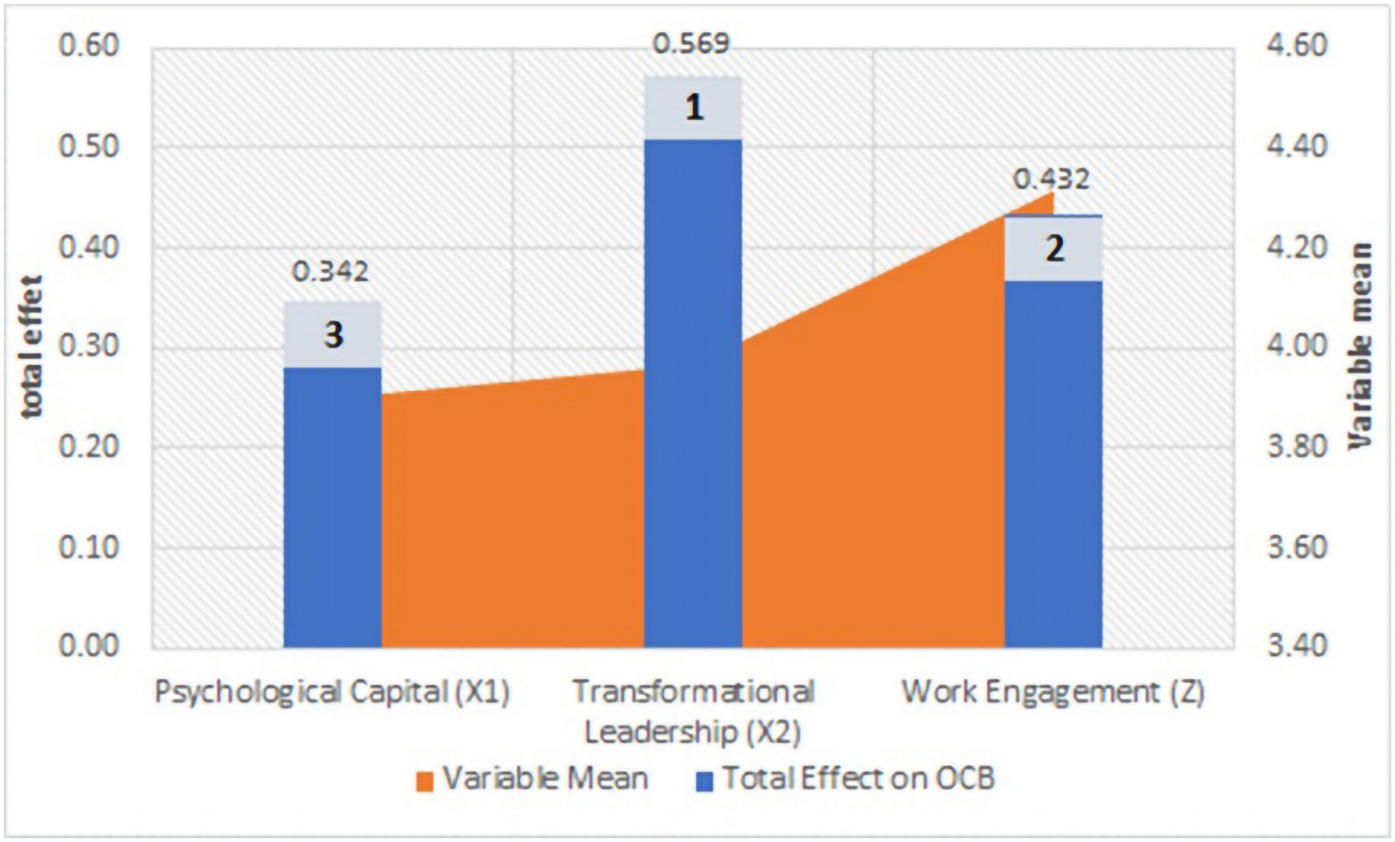
Comparison between the variables’ mean and total effect.

The analysis of the total effect of each variable on OCB is the sum of its direct and indirect effects, the results of which can be seen in Fig 5:

The results of the comparative analysis conclude that for an organization to increase client counselors’ OCB, transformational leadership should be prioritized. Because its direct influence on OCB is high and can substantially affect work engagement as a mediator, the total effect of transformational leadership on OCB goes high. After transformational leadership, the next priority is improved work engagement and psychological capital.

Multigroup analysis was used to analyze the effect between variables based on the gender of the counselors (male and female). The results are summarized in Table 7 below.

**Table 7:** Summary of multigroup analysis.

Structural relationship	Path coefficients		P-value of the difference
	Male	Female	
Psychological Capital → Work Engagement	0.362	0.311	0.657^n.s^
Transformational Leadership → Work Engagement	0.455	0.495	0.728^n.s^
Psychological Capital → OCB	0.274	0.057	0.000**
Transformational Leadership → OCB	0.313	0.554	0.000**
Work Engagement → OCB	0.449	0.295	0.001**

*. Significant at the 0.05 level

**. Significant at the 0.01 level

n.s. Not significant

**Fig 6:**
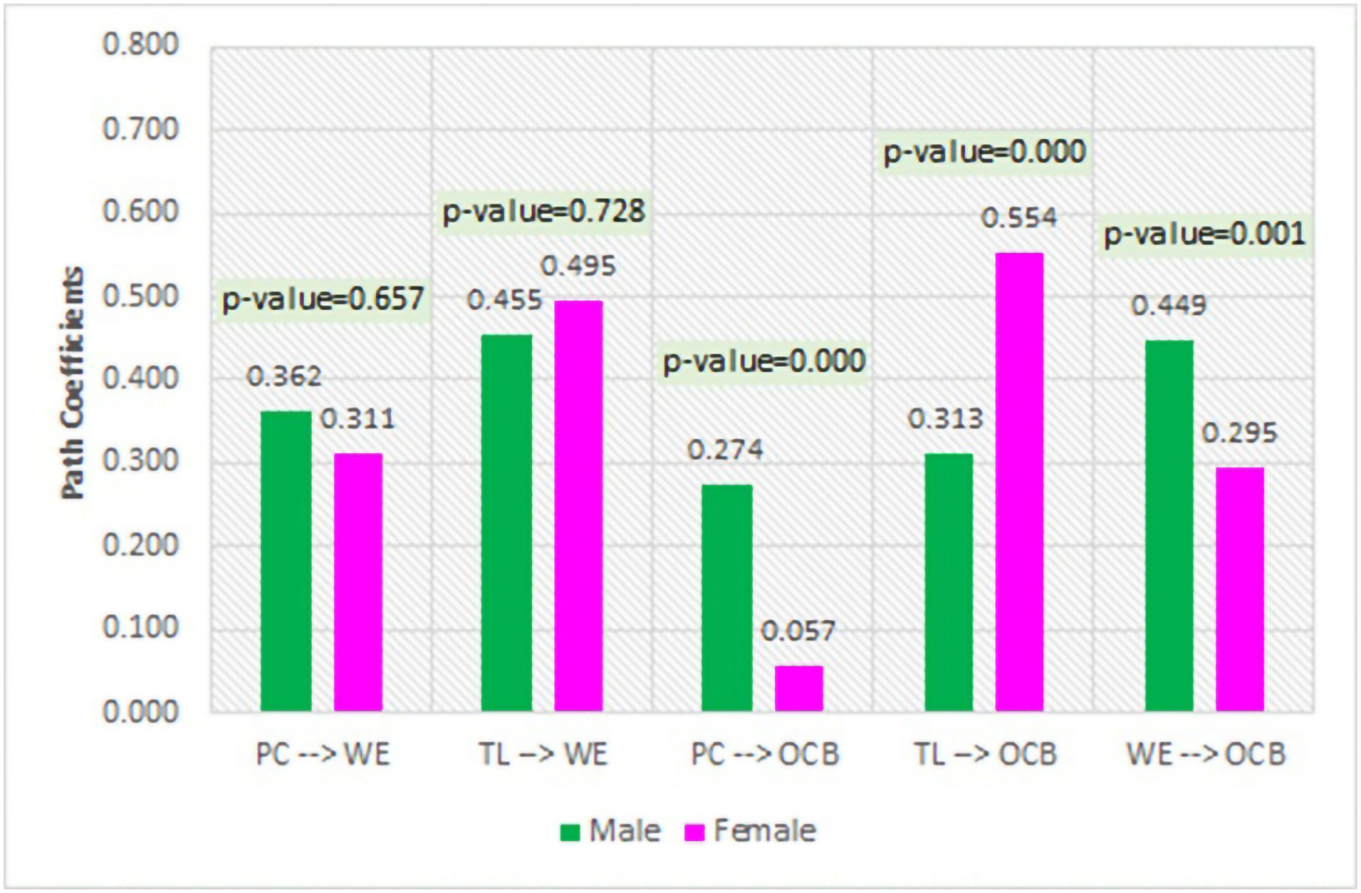
Multigroup analysis based on gender.

The results of the multigroup analysis are depicted in the following figure:

Fig 6 shows the effect of psychological capital and transformational leadership on work engagement in male and female counselors, which statistically has the same effect (p-value>0.05). The difference between male and female counselors can be seen in their effect on OCB. The effect of psychological capital, transformational leadership, and work engagement on OCB among male and female counselors had a significantly different effect value (p-value≤0.05).

In male counselors, OCB is more dominantly influenced by work engagement, while for female counselors, OCB is more influenced by transformational leadership. It provides information that male counselors need more opportunities to be involved in work to increase their OCB, while female counselors need more direction from their leaders.

### 4.2 Discussion

After processing and analyzing the data, it was known that psychological capital significantly affected OCB, which proved that the first hypothesis could be accepted. It showed that counselors with psychological capital could help each other achieve work-related goals that encouraged them to engage in extra-role behavior from OCB. It aligns with previous studies that psychological capital significantly affects OCB [2, 9]. Furthermore, counselors with psychological capital had energy at work, which made them share information with colleagues more freely and easily, leading to positive outcomes such as work engagement. Thus, the second hypothesis, which demonstrated that psychological capital considerably influenced work engagement, could be accepted, as prior research also confirmed that psychological capital positively influences work engagement [8, 28].

This study also proved that transformational leadership is an important driver in fostering positive behavior and learning for counselors leading to OCB. This hypothesis is consistent with earlier empirical studies that demonstrated a positive association between transformational leadership and OCB [12, 20, 21]. In the context of SET, transformational leadership provides positive support to counselors in their work, thereby creating the perception that counselors are obligated to repay this support with positive actions, such as OCB. Furthermore, the study’s results also showed that transformational leadership significantly affected work engagement, which meant that the fourth hypothesis was accepted. Following the previous study’s assertion that transformational leadership influences work engagement, this study’s findings demonstrated that counselors with transformational leadership were more concerned with fostering intrinsic motivation, which had a higher impact on work engagement [11, 17, 24]. In addition, counselors’ work engagement exhibited OCB by being attentive and emotionally invested in their tasks. The fifth hypothesis was therefore accepted. This statement was confirmed by a prior study that work engagement can increase the contribution by doing more OCB [23, 26].

Work engagement strongly mediated psychological capital and transformational leadership on OCB, according to the findings of this study. Thus, the sixth and seventh hypotheses could be accepted and showed partial mediation. It proved that work engagement could encourage them to work harder and help their organization grow by leading to OCB. It was also predicted by the presence of psychological capital and received positive support from the influence of transformational leadership. These findings reinforce the notion that employee qualities in the form of work engagement might mediate the effect of individual and organizational variables on OCB [1, 15].

The results of this study further indicate that there are no significant differences related to variables that affect work engagement in the context of realizing extra roles for counselors. An interesting finding is the differences in the gender of the counselors. The difference between male and female counselors is reflected in the influence of psychological capital, transformational leadership, and work engagement on OCB. It was confirmed that male counselors tend to be more influenced by their work engagement in realizing OCB, which was formed when they worked as counselors. In addition, the results also show that the influence of psychological capital on OCB is more significant in male counselors. In this context, male counselors who are more able to focus, pay attention, and engage in working make them energetic and motivated to take on extra roles. Male counselors engaged in the workplace will exhibit OCB because they feel they can take on additional responsibilities and work quickly.

To summarize, male counselors with high work engagement and psychological capital are more willing to increase their contribution to the organization through OCB. On the other hand, the results of this study indicate that female counselors are more dominantly influenced by transformational leadership in forming OCB. Transformational leadership is known to have the ability to push counselors beyond self-interest in pursuing a shared mission. This finding indicates that female counselors are more influenced to form OCB when the leader gives them awareness and understanding of moral values and inspiration of the right vision to go beyond the goal of the collective good. The transformational leadership effect allows a female counselor to respond quickly to this quality leadership by engaging in OCB. Thus, the results of this study prove that male counselors need more opportunities to engage in work while female counselors need more direction from their leaders in realizing OCB. It can be concluded that male counselors tend to do OCB because of internal factors (psychological capital), while female counselors do OCB because of external factors (transformational leadership).

## 5. Conclusions and implications

### 5.1 Conclusions

According to this study, extra-role conduct, such as OCB, can be accomplished through the influence of psychological capital, transformational leadership, and work engagement. The context of correctional institution as an organization providing public service, which is rarely studied, adds to the originality of this study. Psychological capital and transformational leadership have been shown to impact work engagement and OCB directly. Then work engagement could also mediate psychological capital and transformational leadership towards OCB. This study also demonstrates through multigroup analysis that the effect of psychological capital, transformational leadership, and work engagement on OCB for male and female counselors are statistically varied. In achieving OCB, male counselors are more affected by work engagement, while female counselors are more influenced by transformational leadership. OCB is an action that contributes to the overall organizational effectiveness of an organization [2]. This study proved that counselors with a positive influence from psychological capital, transformational leadership, and work engagement could support the success of OCB, which is beneficial for organizational productivity.

### 5.2 Limitations and suggestions for future research

Future researchers might consider exploring OCB in the wider context of public institutions. First, there are still various antecedents and consequences of OCB that have not been sufficiently researched in public sector organization. Second, it is necessary to conduct studies on OCB in other public institutions, such as the police and firefighters for contextual development of OCB models. Third, future research needs to examine the OCB model with moderation, such as gender which in this study is only used as the basis for multigroup analysis. As for studies on OCB, it can be carried out on a theoretical basis other than SET so that an understanding of OCB in the context of public institutions becomes more comprehensive, such as using Trait Activation Theory to investigate antecedents, and Resource Drain Theory to investigate the consequences of OCB. Next, this study has limitations with using the self-reported questionnaire method in data collection. Future research should use dyadic data collection.

### 5.3 Implications

#### 5.3.1 Theoretical implications

In supporting organizational functions by showing concern for welfare behavior by being selfless through extra roles performed, this study focuses on the role of psychological capital, transformational leadership, and work engagement on OCB in the context of public institutions. The role of psychological capital and transformational leadership are exogenous variables that can positively impact OCB. Psychological capital is a personal resource that encourages counselors to have significant psychological well-being leading to extra behavior. Additionally, transformational leadership can push employees beyond self-interest and reasonable cause to improve OCB as a reciprocal of behavior for their leaders’ treatment. This research found that work engagement can also become a positive role appropriately and be used as an intervening variable. Employees with work engagement are more likely to focus and get emotionally involved with work, making them more likely to leave their formal roles actively. It builds on the previous findings regarding the role of work engagement as a mediator that contributes to OCB, which is still scarce in the context of public institutions [15]. Thus, this research can provide insight that if an individual has psychological capital influenced by transformational leadership, and shows work engagement, then the realization of extra-role behaviors such as OCB would be encouraged.

#### 5.3.2 Managerial implications

The findings of this study may be used by organizational management to provide suggestions regarding the role of psychological capital and transformational leadership in influencing OCB, as well as the significance of work engagement in mediating this connection. According to the link between the factors, it is possible to influence constructive psychological changes, manifesting as positive behavior. Practically, organizations must take into account good psychological capital in the recruitment process. Psychological capital assessment needs to be adopted as one of the elements in the employees’ selection process. With good psychological capital conditions from the start, organizations can manage individuals more effectively even when faced with hardship.

Additionally, organizations must establish transformational leadership features as prerequisites for occupying organizational leadership positions in order to ensure that leaders manage individuals in a transformative manner. To ensure the sustainability of transformational leadership, however, organizations must regularly assess the transformational actions of leaders in managing their subordinates. In addition, this evaluation must be accompanied by a just reward and punishment system. As part of their regeneration efforts, organizations must also conduct transformational leadership training and development in order to prepare potential transformative leaders.

With these efforts, the organization is expected to be able to encourage employees to pursue objectives by being willing to spend effort and persevere with their tasks, and inspire them to modify their views and skills about performance improvement beyond personal interests for the organization’s advantage. Thus, OCB will develop into an activity that employees engage in freely via behavior that transcends the formal requirements of their position and adds to the organization’s effective operation. Implementing OCB can be done in a different way or process.

Gender differences can impact the likelihood of counselors forming an OCB, although other factors are considered more influential. The male counselors require psychological capital to produce stronger positive emotions. In addition, when male counselors are enthusiastic about their work, they often take on more responsibilities. On the other hand, female counselors favor leadership that can inspire them to modify their attitudes, beliefs, talents, and motivations for the organization’s benefit. Consequently, female counselors may instill OCB with a sense of self-motivation, allowing them to join voluntarily. To effectively implement OCB, organizational management must encourage workers to demonstrate attitudes beyond the job description, which cannot be accomplished only through rewards. OCB may be achieved for an organization’s success if individuals’ intrinsic impact drives them to enjoy performing work outside of their job description willingly.

## Supporting information

S1 Data (DOCX)
