# Supplementary material for: Do psychological capital and transformational leadership make differences in organizational citizenship behavior?
Source: PLoS One. 2023 Dec 15;18(12):e0294559. doi: 10.1371/journal.pone.0294559 (PMC10723732; doi:10.1371/journal.pone.0294559)
Supplement: S1 Data — (DOCX) [file pone.0294559.s001.docx]

**Suplementary Data**

<https://doi.org/10.1016/j.dib.2022.108243>
